# Popliteal Aneurism

**Published:** 1862-06

**Authors:** 


### MERCY HOSPITAL.

Service Prof. J. H. HOLLISTER, Attending Surgeon.

### POPLITEAL ANEURISM.

Reported for the EXAMINER.

GENTLEMEN:—We have before us a case of interest for examination, demanding an immediate operation; but before proceeding with it, I propose to call your attention to the location of the tumor; the enormous size to which it has attained; and ask you, by careful manipulation, to educate your fingers to the detection of the *aneurismal thrill.* The books describe to you two kinds of aneurism, *true* and *false*, and point out with sufficient clearness the distinctive characteristics of each. They also remind you of the liabilities to a mistaken diagnosis of tumors, not aneurismal, from their proximity to, and impulse from, large arteries in immediate relation. That the tumor before us is aneurismal is evident, from the fact that when you fully control the femoral artery, not only does the *thrill* cease to be felt, but by gentle compression the tumor rapidly diminishes in size, and the walls, before tense and nearly unyielding, become soft and even flabby.

This is termed a popliteal aneurism from being developed in the popliteal space upon the popliteal artery; and this variety is of much more frequent occurrence than any other. The size to which this has attained is very unusual. You will notice that it has borne before it and moulded itself around both the internal and external flexor tendons, and so fully occupied the popliteal space as to permit but a very limited flexion of the knee-joint.

Measured around its largest development, the limb is $18\frac{3}{4}$ inches in diameter, while the other, at the corresponding point, measures only 12 inches; from above downward, the measurement of the tumor is 8 inches; while the thrill may be detected from its extreme inner to its outer margin for a distance of 12 inches. It will doubtless surprise you to notice what an impulse is given to this enormous sac, by the pulsation of the popliteal artery, and you will more fully than before comprehend the power of the heart-impulse to the circulation.

With a degree of care compatible with the patient's comfort, I desire each member of the class to study the case, noting the absence of the thrill, as with compression upon the femoral artery, you control the circulation, and the immediate subsidence of the tumor, showing that its contents—probably not less than two pints—is almost entirely fluid. While you are conducting this examination individually, and before administering the anœsthetic, I will briefly detail to you the previous history of the case.

The patient, F. G. aged 40, a native of England, 6 feet and 1 inch in height, and of spare habit, was admitted to the Hospital about a week since, having been discharged from army service, with the desire to be cured by such means as we might deem most safe, and at the same time permanent.

He has suffered severely from the constitutional effects of *syphilis*, and at the same time presents the anemia incident to the excessive use of *alcohol*. These, gentlemen, are the two fruitful causes of aneurism when not traumatic.

Fearing, as the result of ligation, the refusal of the wound to heal kindly, and the chances of secondary hemorrhage, I placed him at once upon full diet, with tonic treatment, and applied along the femoral artery, at four different points, successively, the horse-shoe tourniquet, with results which you here behold.

Although adjusted with the utmost care, to prevent unnecessary irritation, the compress could not be applied for eight hours with sufficient force to even partially control the artery, without producing, first, serous effusion, second, erysipelatous inflammation of the skin, and third, gangrene at the precise point of pressure. Unfavorable as the case really may be, there is no time to be lost; the last hope of the man depends upon the immediate ligation of the artery, for the tumor upon its lower prominence has already, by pressure, stopped the circulation in the sack and investing skin; gangrene of the central portion has already supervened; and in less than 36 hours, without ligation, the sac will doubtless be ruptured, and the hemorrhage fatal, unless controlled.

I should have remarked, with reference to the development of this aneurism, that for six months after its first appearance, up to Dec. 1, 1861, it was about the size of a filbert, with no appreciable change in size. At that time it began rapidly to enlarge, and in a few days attained to nearly its present size.

As soon as the patient is sufficiently insensible, I shall ligate the femoral, about two inches below the origin of the profunda, for the reason that the artery is here most superficial, and I desire as little as possible to increase the chances of sloughing, by reason of dissection. Before I proceed to the incision, I have to remind you of the cautions enjoined by all writers with reference to disturbing the femoral vein, with the danger of laceration or phlebitis, and also to avoid including the nerve in the ligature. Having opened the sheath of the vessels, and raised the artery alone upon the hook, armed with the ligature, I pause a moment to determine if, by proper tension, the thrill is controlled in the sac, for *sometimes* we have here duplicate arteries, and must be sure that we do not fail of our intention to starve out this tumor, by entirely withholding its supplies.

Having ligated the artery, it remains to dress the wound, secure its rest, sustain the patient, and wait, I confess with not very sanguine expectation, the development of secondary results. Among the more important of these results which we have to anticipate, are inflammation, especially of an erysipelatous character;—death of the limb from deficient supply of blood; and secondary hemorrhage when the ligature finally cuts through the artery. In a patient enjoying good health up to the time of the operation, it would generally be a sufficient safeguard against these anticipated evils, to place the limb in an easy relaxed position, the leg and foot being covered with warm, dry flannel; to give a full anodyne internally, to be followed by a light diet and cool drinks; and to keep the temperature of the room between 60° and 65° of Fahrenheit. But the extremely unfavorable constitutional condition of the patient before us; and especially the indications already given of a tendency to gangrenous erysipelas, make the vigilant employment of other prophylactic measures necessary. Among the most efficient agents for this purpose is the tincture of chloride of iron, given in full doses internally. Hence, in addition to the anodynes necessary to procure rest, we shall give 20 gtts. of this preparation of iron every four hours, and as much nutritious food as his stomach will bear. Owing to his previously intemperate habits, it may become necessary to resort, temporarily, to the use of alcoholic stimulants, and if so, they will be given with sweet milk, in the form of punch, that nutritious matter may accompany the stimulant into the blood. Independent of all moral considerations, however, there is a strong objection to the use of alcoholic stimulants in the treatment of aneurismal patients, either before or after an operation. You will find almost all modern authors speaking of an atheromatous or fatty degeneration of the coats of the arteries as one of the causes of spontaneous aneurisms. And such a condition of the coats of the vessel, also, adds greatly to the danger of secondary hemorrhage after the application of the ligature. Very few pathological facts are better established, than that the free use of alcoholic stimulants favors fatty degeneration in nearly all the fibrous tissues, and in none more than in the heart and larger blood-vessels. Hence, they are contra-indicated in aneurisms.

Your attention is directed thus minutely to the dangers that threaten the patient, and the means for averting them, because the success of surgical operations often depend more on the preceding and subsequent treatment, than on the skill with which the operations are performed.

June 9, 1862.—Five weeks have now intervened since the operation. The patient was for sometime extremely prostrated, not alone from the operation, but from the gradual withdrawal of the stimulants, from the inordinate use of which, he was quite on the verge of *tremens*, when he entered the Hospital. The ligature came away the 24th day without hemorrhage, and the wound healed well. The sloughs from compression have been slow in separating, and the resulting ulcers became filled with abundant false granulations, but are now nearly healed. The slough resulting from the gangrenous spot on the under surface of the aneurismal tumor did not separate until about one week since, when a fetid sanious discharge of two or three ounces occurred.

During this period the tumor had diminished in size about one-third; but is still fluctuating, and from the nature of the discharge, we evidently have nothing to hope from the *fibrination of the clot.* Indeed, during the last three or four days the patient has become more feeble, the pulse quicker and weaker, and the countenance sunken. It is thus very evident that instead of *fibrination* and gradual contraction of the clot, it has become partially disorganized and putrid in the sac, and is exerting an injurious influence on the blood and vital properties of the patient. On examination, the opening into the sac was found to be large enough to admit the index finger, with which the clot was thoroughly broken up, and discharged to the amount of nearly eight ounces. The flabby walls of the extensive cavity thus left were supported by a bandage, and directions given to have the patient vigorously supported by quinine and iron, and the sac to be carefully washed out once a day, with a solution of one grain of chloride of zinc to the ounce of water.

June 16, 1862.—During the past week, the patient has improved rapidly in all respects. He is able to go about his room; the cavity in the popliteal space is very much diminished; and the discharge from it is small in quantity and healthy in quality. His recovery may be considered complete.

